# Acute kidney injury as a severe complication of diabetic ketoacidosis in children: a case report

**DOI:** 10.1186/1687-9856-2013-S1-P6

**Published:** 2013-10-03

**Authors:** Faisal Bukkar, Novina Andriana, RM Ryadi Fadil, Ahmedz Widiasta

**Affiliations:** 1 Department of Child Health, Faculty of Medicine, Universitas Padjadjaran, Dr.Hasan Sadikin General Hospital, Bandung, Indonesia

## Background

Diabetic Ketoacidoasis (DKA) is associated with significant morbidity and mortality and probably could be prevented by earlier diagnosis of diabetes mellitus (DM) and intervention. The level alertness of primary care doctors and knowledge on DM play a role to prevent severe complication of DKA.

## Objective

To highlight the acute kidney injury as a severe complication of DKA in children.

## Methods

We reported a diabetic ketoacidosis patient developing acute kidney injury at Hasan Sadikin General Hospital Bandung, Indonesia.

## Results

A 12-year-old girl from a rural area was admitted to our pediatric emergency with decreased consciousness. We reviewed there was a history of polyuria, polydipsia and marked weight loss. When she arrived at hospital, she was very ill, comatose state, severe dehydration and typical Kussmaul breathing. Her heart rate was 140/min with thread pulse, low blood pressure, dry mucous membranes, sunken eyes, poor capillary return, and cold fingers. Laboratory analysis showed her blood glucose level was 890 mg/dl, severe metabolic acidosis and urine ketones 3+. The patient was resuscitated with iv fluid as soon as possible, followed by insulin and potassium chloride. On day 2 she developed oliguric and her serum creatinin and urea levels were 2.25 and 124 mg/dl that impressed as acute kidney injury. After fluid restriction she had persistent oliguric, increased serum creatinin and urea up to 7.34 and 234 mg/dl warranted initiation of peritoneal dialysis. After peritoneal dialysis and DKA management she showed a good improvement.

## Conclusion

Patient with severe DKA developing acute kidney injury need early recognition and initiating renal intervention may improve the potentially poor outcome.

